# IE-IQA: Intelligibility Enriched Generalizable No-Reference Image Quality Assessment

**DOI:** 10.3389/fnins.2021.739138

**Published:** 2021-10-21

**Authors:** Tianshu Song, Leida Li, Hancheng Zhu, Jiansheng Qian

**Affiliations:** ^1^School of Information and Control Engineering, China University of Mining and Technology, Xuzhou, China; ^2^School of Artificial Intelligence, Xidian University, Xi'an, China; ^3^Pazhou Lab, Guangzhou, China; ^4^School of Computer Science and Technology, China University of Mining and Technology, Xuzhou, China

**Keywords:** image quality assessment, NR-IQA, intelligibility, distortion, generalization, semantic

## Abstract

Image quality assessment (IQA) for authentic distortions in the wild is challenging. Though current IQA metrics have achieved decent performance for synthetic distortions, they still cannot be satisfactorily applied to realistic distortions because of the generalization problem. Improving generalization ability is an urgent task to make IQA algorithms serviceable in real-world applications, while relevant research is still rare. Fundamentally, image quality is determined by both distortion degree and intelligibility. However, current IQA metrics mostly focus on the distortion aspect and do not fully investigate the intelligibility, which is crucial for achieving robust quality estimation. Motivated by this, this paper presents a new framework for building highly generalizable image quality model by integrating the intelligibility. We first analyze the relation between intelligibility and image quality. Then we propose a bilateral network to integrate the above two aspects of image quality. During the fusion process, feature selection strategy is further devised to avoid negative transfer. The framework not only catches the conventional distortion features but also integrates intelligibility features properly, based on which a highly generalizable no-reference image quality model is achieved. Extensive experiments are conducted based on five intelligibility tasks, and the results demonstrate that the proposed approach outperforms the state-of-the-art metrics, and the intelligibility task consistently improves metric performance and generalization ability.

## 1. Introduction

Image quality assessment (IQA) plays a vital role in image acquisition, compression, enhancement, retrieval, etc. The existing IQA metrics are mainly designed for synthetic distortions and cannot be applied to wild images satisfactorily due to the limited generalization ability. Fundamentally, image quality embodies two aspects: distortion and intelligibility (Abdou and Dusaussoy, 1986). Most IQA algorithms only focus on the distortion measurement and the intelligibility aspect is rarely investigated. In this paper, we mainly investigate the role of intelligibility in building a highly generalizable IQA model.

Intelligibility refers to the ability of an image to provide information to a person or a machine (Abdou and Dusaussoy, 1986), that is, the degree to which the image could be understood. Distortions affect image intelligibility, and accordingly, intelligibility is indicative of image quality when humans make judgments. Traditional handcrafted feature-based IQA metrics mainly focus on distortions and cannot commendably describe image intelligibility. Deep learning-based methods learn the IQA task in a data-driven manner, and consequently do not directly pay attention to image intelligibility, either.

**Figure 1:**
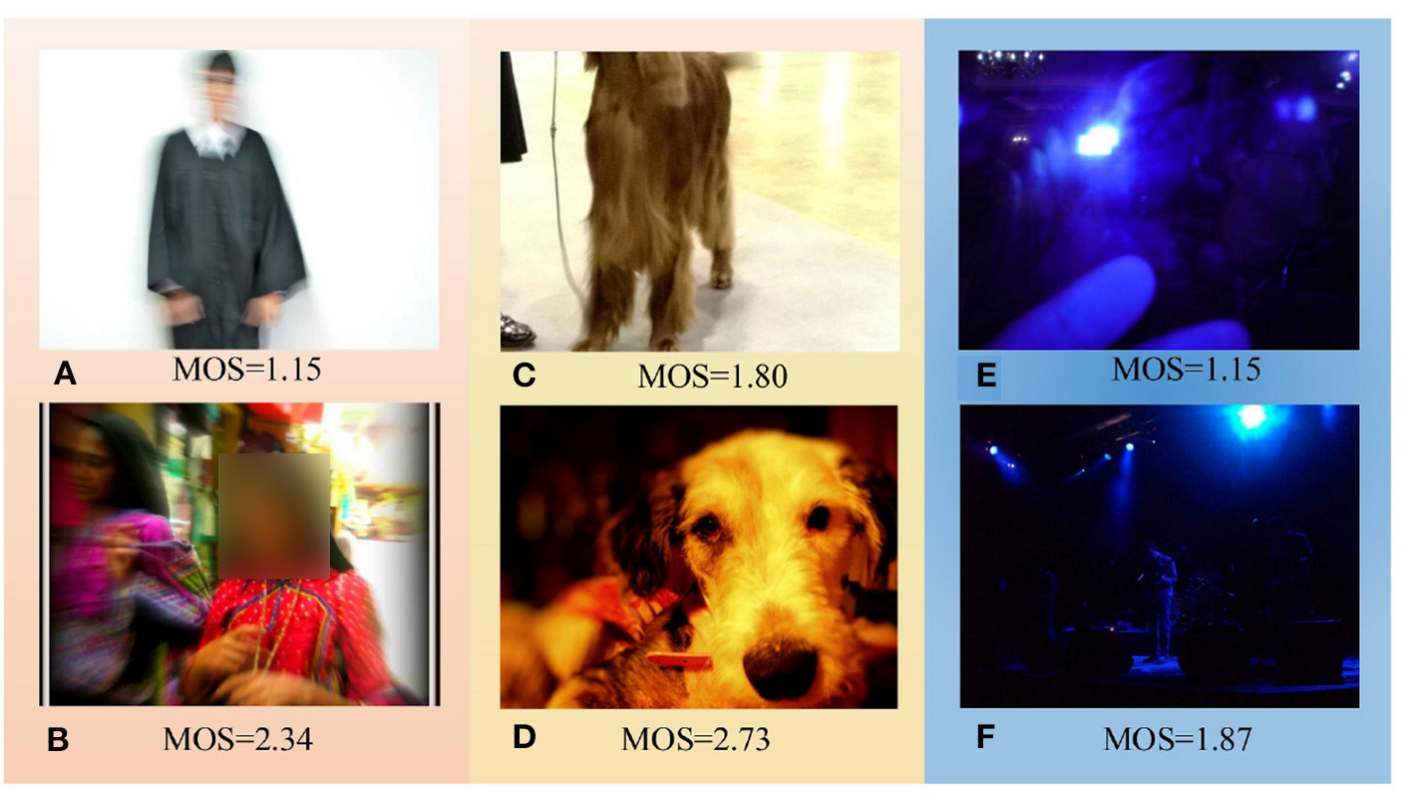
Relation between intelligibility and image quality. **(A–F)** Compared to images in the first row, images in the second row have higher intelligibility and accordingly higher mean opinion score (MOS). Images are from the KonIQ-10k (Hosu et al., 2020) dataset. The range of MOS is [1, 5], and higher MOS means better quality.

Since the most essential function of image is to convey information, when distortions seriously undermine the expression of information, the intelligibility will also become low, which in turn indicates poor image quality. Real-world images are typically contaminated by complicated distortions, which lead to different degrees of intelligibility. Figure 1 explains how intelligibility indicates image quality. Figures 1A,B both suffer from severe motion blur, and both contain human as the main content. The human face in Figure 1A is too blurred to be recognized, whereas a woman's face in Figure 1B can still be easily identified. Thus, Figure 1B has higher intelligibility and accordingly higher quality score. The distortion in Figure 1C is not heavier than Figure 1D, but Figure 1D is easier to be recognized; hence, Figure 1D has higher intelligibility and accordingly higher quality score. Finally, Figures 1E,F was mainly underexposed with locally overexposed. The main content in Figure 1E is illegible, whereas Figure 1F can still be distinguished as a singing stage with performers. Therefore, the quality of Figure 1F is better than that of Figure 1E. It can be concluded from Figure 1 that images with similar distortions may have significantly different quality due to different degrees of intelligibility. Therefore, a robust quality assessment metric should also take intelligibility into account, especially for severe distortions.

Motivated by the above facts, this paper presents a new framework to achieve highly generalizable image quality assessment by integrating intelligibility and distortion measure. The intelligibility of an image can be represented from different perspectives, such as “whether the content of the image is recognizable,” “which category does the main object in the image belong to,” and “what scene does the image show.” The results of these questions are all important information conveyed by the image, and through the mining of these questions, we can obtain descriptions of image intelligibility. These questions can be described by popular computer vision tasks, such as image classification, scene recognition, object detection, and instance segmentation. Therefore, we calculate intelligibility features based on these semantic tasks. Then, we propose a bilateral network to combine the distortion features and intelligibility features. Further, we design different feature selection strategies for different semantic understanding tasks. This produces highly generalizable intelligibility features. The distortion network is applied to extract distortion features that are complementary to those intelligibility features. With the bilateral network, highly generalizable intelligibility features with rich semantic information can be fused with distortion features, producing the final IQA model.

The contributions of this work are summarized as follows:

- We propose a new framework for designing highly generalizable image quality models by integrating intelligibility and distortion, two fundamental aspects of image quality. In the proposed framework, intelligibility features can be extracted based on popular semantic tasks, such as image recognition, scene classification, and object detection.
- We propose a bilateral network with an intelligibility enhanced module to fuse intelligibility features with distortion features for building a robust IQA model. A feature selection strategy is proposed to extract intelligibility features instead of doing direct training. This strategy can avoid the risk of damaging generalizable features.
- We have verified the effectiveness of the proposed method through extensive experiments and compared with the state-of-the-arts. The experimental results demonstrate that the proposed model can achieve significantly better generalization performance.

## 2. Related Work

Early no-reference IQA (NR-IQA) metrics typically train a regressor to obtain quality scores based on handcrafted features. For example, BLIINDS-II (Saad et al., 2012), BRISQUE (Mittal et al., 2012), and BIQI (Moorthy and Bovik, 2010) designed features meticulously through natural scene statistics (NSS). NFERM (Gu et al., 2014) incorporated features inspired by the free energy theory, human visual system, and NSS. CORNIA (Ye et al., 2012) and HOSA (Xu et al., 2016) trained large-scale visual codebooks from natural image to make predictions. The above handcrafted feature-based IQA models are usually limited in handling the diversified scenes and distortion types in real-world images.

With the boom of deep learning, convolutional neural networks have been widely applied in IQA. Early attempts utilized relatively shallow networks (Kang et al., 2014; Kang et al., 2015; Kottayil et al., 2016) to extract features for assessing synthetic distortions. Then, deeper networks were utilized to handle more complex distortions (Bosse et al., 2017; Kim and Lee, 2017; Ma et al., 2017; Yan et al., 2019; Zhai et al., 2020; Zhang J. et al., 2020). It is widely acknowledged that large datasets are needed for training deep neural networks. However, so far the largest IQA dataset only has 11,125 images, which are still limited. Thus, recent deep IQA metrics (Bianco et al., 2018; Varga et al., 2018; Zhang W. et al., 2020) utilize networks pre-trained on large-scale computer vision tasks and then fine-tune on them. For example, Bianco et al. (2018) made fine-tuning on the model pre-trained on subset of ImageNet (Imagenet large scale visual recognition challenge, 1.3M images) (Russakovsky et al., 2015) and Places-205 (Wang et al., 2015) (2.5M images). Varga et al. (2018) made fine-tuning on deep pre-trained network (ResNet101 He et al., 2016) to learn the distribution of mean opinion score (MOS). Zhang W. et al. (2020) utilized two different networks to evaluate synthetic and authentic distortions, respectively, and the authentic network was fine-tuned on pre-trained network (VGG16, Simonyan and Zisserman, 2015). Make fine-tuning on pre-trained model of recognition task is a suboptimal method because IQA task is different from recognition tasks. Recognition tasks should be robust to distortions while IQA should distinguish distortions. Though fine-tuning with IQA images can improve IQA performance, the generalizable features trained with large-scale dataset were damaged during further training. And due to the small sample property of IQA, generalization ability of new features is still unsatisfying and cannot be adopted to real-world applications.

Until recently, the generalization problem of IQA models began to receive attention. Zhu et al. (2020) adopted meta-learning to learn the prior knowledge of distortions in synthetic distortions and then fine-tune on authentic distortions to achieve better generalization ability. Hosu et al. (2020) built a large dataset (KonIQ-10k: 10,073 images) for model training and obtained better generalization performance. Su et al. (2020) incorporated semantic features and multi-scale content features to handle challenges of distortion diversity and content variation. The above methods have achieved better generalization performance than earlier metrics, but their generalization ability is still far from ideal and further explorations are needed. In this paper, we work toward this direction by proposing a new framework to address the generalization problem, where the intelligibility property of images is investigated.

## 3. Proposed Method

### 3.1. Relation Between Intelligibility and Quality

As aforementioned, image intelligibility can be described by semantic understanding tasks. The most popular one is the classification task on Imagenet Large Scale Visual Recognition Challenge, which has 1.3 M images belonging to 1,000 classes (Russakovsky et al., 2015). Therefore, we take the deep convolutional neural network (DCNN) trained on this task as an example. The output of the classification network is a probability distribution *o*_*i*_, *i* = 1, 2, ..., 1, 000, and 1, 000 is the total number of classes. The prediction confidence *c* can be obtained by


(1) $$\begin{array}{r} c=max\left(o_{i}\right),i=1,2,...,1000. \end{array}$$

The confidence *c* in Equation (1) also represents the top1-probability. If the intelligibility of an image is high, the model may easily recognize the category and the top1-probability may be notably high. When the intelligibility is low, the model will be unconfident of its predictions and the top1-probability also tends to be low.

**Figure 2:**
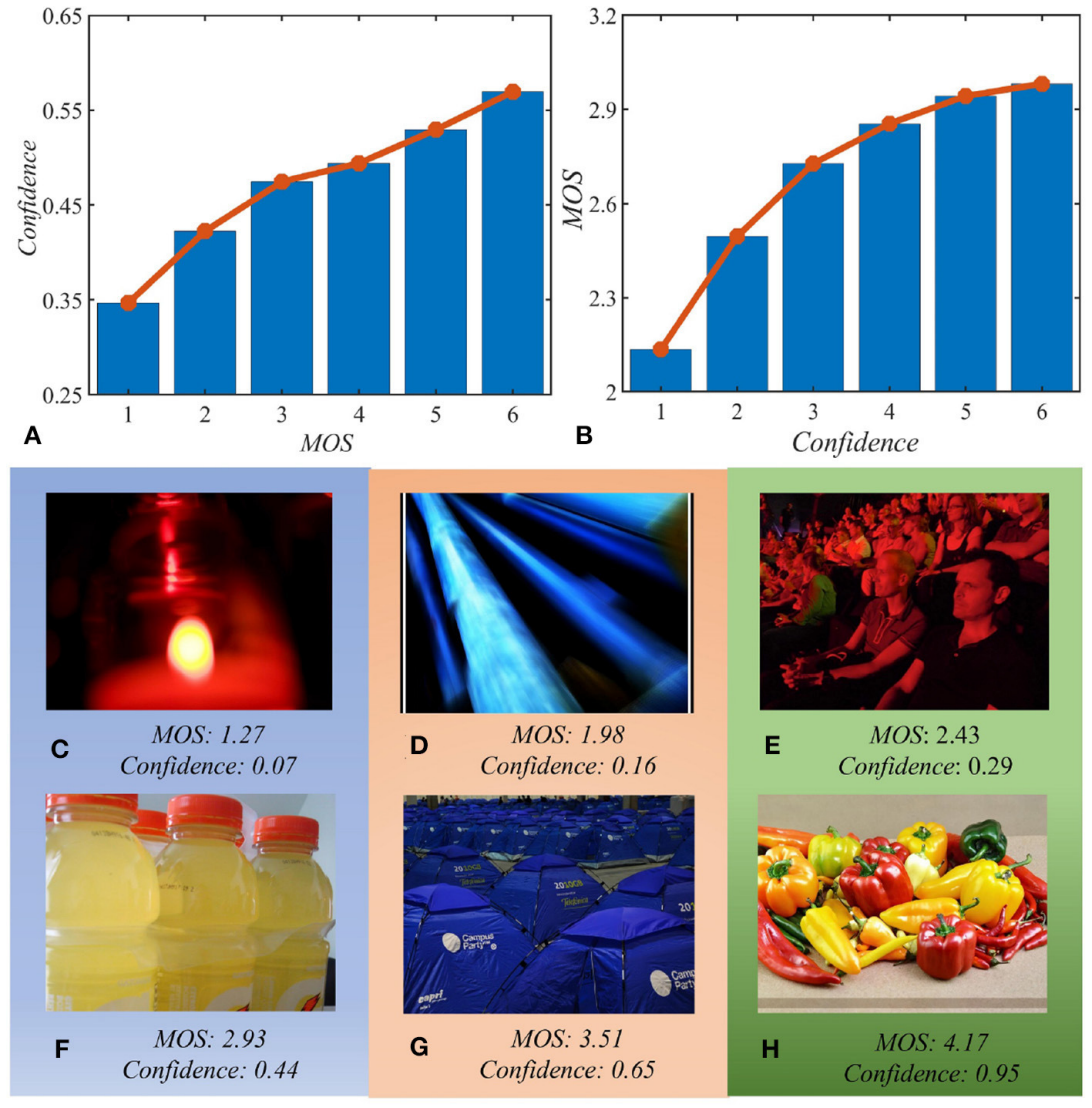
Relation between recognition confidence and image quality. **(A)** Image quality affects recognition confidence; **(B)** recognition confidence indicates image quality; **(C–H)** representative images with different prediction confidence and mean opinion score (MOS). Panels **(C–H)** correspond to six ascending bins of B whose MOS increases with confidence. All results are obtained from the KonIQ-10k dataset based on EfficientNet-B0 network (Tan and Le, 2019).

To have an intuitive understanding of the above characteristic, we compare the average classification confidence score obtained from images of different quality. First, we divide images from an IQA dataset into several groups according to their MOS values in ascending order. (Specifically, MOS are divided into 6 equal intervals of [*m*_*i*_, *m*_*i*+1_] where *i* = 1 − 6, *m*_1_ = *min*(*MOS*), *m*_7_ = *max*(*MOS*).) Then, we utilize an image classification model trained on ImageNet to obtain the confidence score of images in each group. Finally, we calculate the average confidence score of each quality group, and illustrate them in Figure 2A. We can observe that images with poor quality tend to have lower prediction confidence than those with high quality. That is, image quality does have a significant impact on intelligibility.

In this paper, we are more interested in how intelligibility indicates image quality. Therefore, we do another experiment by dividing images according to the prediction confidence and compare the average MOS value of different confidence intervals. The results are presented in Figure 2B. Furthermore, to show the relation more intuitively, we also show sample images in Figures 2C–H that corresponds to the six ascending bins of Figure 2B. We can observe from Figures 2B–H that intelligibility described by image recognition task can distinctly indicate image quality.

### 3.2. Our Framework

**Figure 3:**
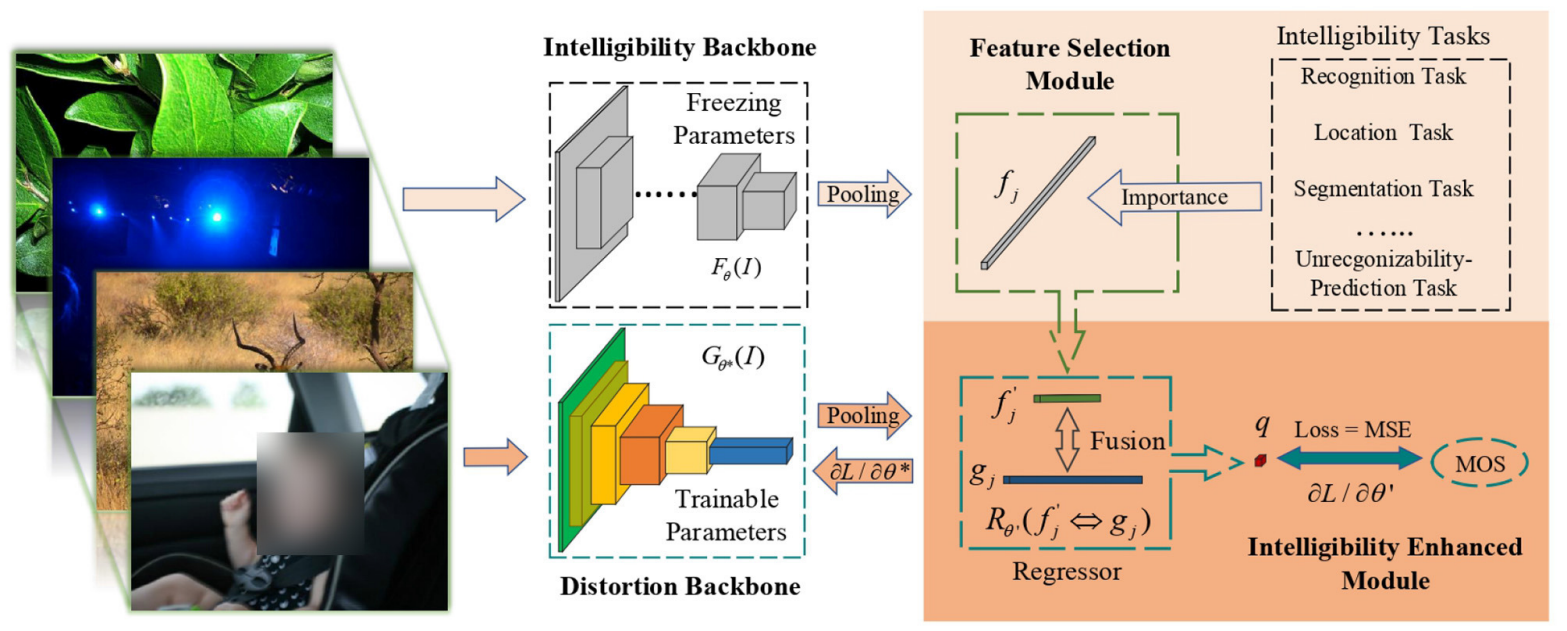
Proposed framework of IE-IQA. Our framework contains intelligibility and distortion backbone, and colorful blocks in the distortion backbone are trainable while gray blocks in the intelligibility backbone are not trainable. An intelligibility enhanced module is adopted to fuse distortion features with intelligibility features obtained from the proposed feature selection module.

In this paper, we propose an intelligibility enriched IQA (IE-IQA) framework, as illustrated in Figure 3. In our framework, we propose a bilateral network to integrate intelligibility features and conventional distortion features. Since intelligibility can be represented using different image understanding tasks, it is reasonable to utilize features from these tasks as intelligibility features. However, IQA is different from image understanding tasks, and directly utilizing features of image understanding tasks may lead to negative transfer, which has been proved by many transfer learning researches (Pan and Yang, 2010; Cao et al., 2018; Zhang J. et al., 2018). Since intelligibility is vital to our framework, utilizing features that are most relevant to intelligibility is a better way. Thus, we first propose a feature selection module to pick out more relevant features, and then fuse them with distortion features through an intelligibility enhanced module.

The distortion backbone with parameter θ^*^ in Figure 3 is denoted as $G_{\theta^{*}}$, which is adopted for extracting distortion features *g*_*j*_ from image *I*. The intelligibility backbone *F*_θ_ with parameter θ is adopted for extracting intelligibility features *f*_*j*_. We select the most important features $f_{j}^{\prime }$ from *f*_*j*_ and then fuse them with distortion features *g*_*j*_ (denoted as $f_{j}^{\prime }\Leftrightarrow g_{j}$) to obtain quality score *q* through a regressor $R_{\theta^{\prime }}$. The whole process is explained as follows:


(2) $$\left\{ \begin{array}{l} \;f_{j}=F_{\theta }(I),\\ \;f_{j}^{\prime }\leftarrow f_{j},\\ \;g_{j}=G_{\theta^{*}}(I),\\ q=R_{\theta^{\prime }}(f_{j}^{\prime }\Leftrightarrow g_{j}). \end{array}\right.$$

In this paper, four extensively studied semantic understanding tasks are utilized to obtain intelligibility features, including image recognition on subset of ImageNet (Russakovsky et al., 2015), scene classification on Places-365 (Zhou et al., 2017), object detection and instance segmentation on MS-COCO (Lin et al., 2014). In addition, we also utilize a relevant unrecognizability prediction task, which predicts the unrecognizable degree of an image. This task is trained on the VizWiz-QualityIssues dataset (Chiu et al., 2020), containing images with labels of the unrecognizable degree. Even if intelligibility features of heavily distorted images cannot obtain desired results in original tasks, they can still be distinguished from features of high-quality images, which is beneficial to the IQA task.

In our framework, the distortion backbone works in a data-driven manner to search for the best distortion features, and the intelligibility backbone is guaranteed to obtain features with high generalization ability and rich semantic information. To achieve these goals, we propose to freeze parameters θ of the intelligibility backbone during the training process while keeping parameters θ^*^ in the distortion backbone trainable. On the one hand, the distortion network loads the pre-trained model trained on ImageNet. Though the pre-trained model has decent generalization ability, we still need to train the feature extractor with image quality data so that the network can adapt to IQA task and obtain better performance. Therefore, we make parameters of the distortion backbone trainable. On the other hand, training the intelligibility backbone may be problematic. High level features of image understanding tasks are rich in semantic information which is generalizable. If we train the intelligibility network using the IQA data, the generalization ability of intelligibility features (which are already generalizable) may be destroyed. Therefore, we freeze the intelligibility backbone to handle this problem.

In the proposed intelligibility enhanced module, we tried several feature fusion strategies: (1) utilize one/two/three fully connected (FC) layers to regress the quality score and fuse intelligibility features to different FC layer with add/multiply/concatenate operation; (2) utilize other layers to align intelligibility features with distortion features and then use other FC layers to regress the quality score; (3) utilize auxiliary layers and loss fuction to train intelligibility features along with strategy-(1) or strategy-(2); (4) replace low-dimensional features with sparse selected features (features that are not selected are set to zero) and then utilize strategy-(1) or strategy-(2). In implementation, we have found that these strategies achieve similar results. Due to the feature selection module, it is easy to combine lower dimensional intelligibility features and simple strategy can obtain satisfying results. The loss function we utilized is the mean square error (MSE).

### 3.3. Feature Selection

During the feature fusion process, we propose strategies to select intelligibility features. For a specific semantic understanding task, only a part of neural units and corresponding features in a DCNN are significantly activated during the inference process, while others are not vital to the final prediction and intelligibility (Hu et al., 2016; Zhang Q. et al., 2018; Zhou et al., 2019). Since introducing too many features are not conducive (even harmful in many transfer learning experiments) to IQA performance and generalization, we design feature selection strategies for different tasks based on contribution and sensitivity. Contribution-based strategy chooses features with greater contributions to predictions while sensitivity-based strategy chooses features that predictions are more sensitive to.

#### 3.3.1. Contribution-Based Strategy

We propose to select features that have prominent contributions to final predictions. Theoretically, this strategy is not limited to any specified network as long as the network can be separated into a backbone and one FC layer. In fact, this kind of network architecture is very common in the image classification and scene recognition. Specifically, the output of backbone can be denoted by *f*_*j*_, *j* = 1, 2..., *N*_*d*_, where *N*_*d*_ is the dimension of features and the output-logits of the FC layer can be described by


(3) $$\begin{array}{r} z_{i}=\overset{N_{d}}{\underset{j=1}{\sum }}w_{ij}\times f_{j}+b_{i}, \end{array}$$

where *w*_*ij*_, *b*_*i*_, *z*_*i*_ are weights, bias and logits of the FC layer, *i* = 1, 2, ..., *C*, and *C* is the number of total classes. The feature selection strategy is shown in Algorithm 1. In Algorithm 1, we locate the top1-probability first. Then, we calculate the contribution of each dimension of feature *f*_*j*_ by


(4) $$\begin{array}{r} contrb_{j}=abs\left(w_{i_{max},j}\times f_{j}\right). \end{array}$$

**Algorithm 1:**
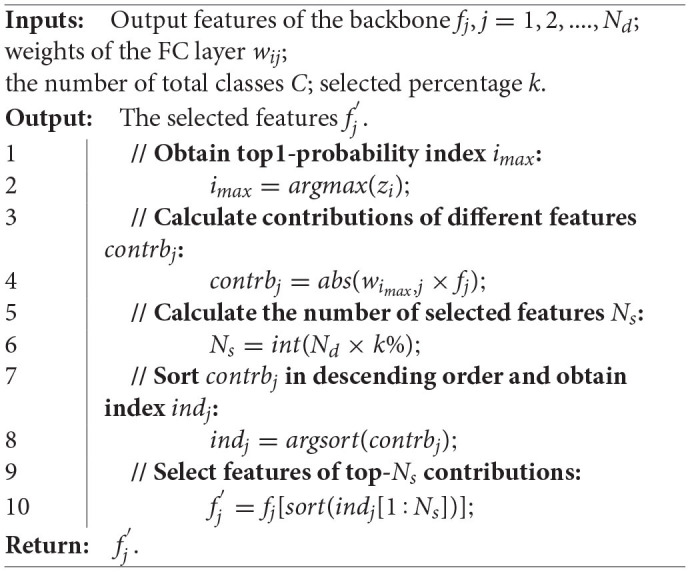
Feature selection strategy based on contributions.

In Equation (4), the contributions of features are determined by both weights and activation values. Finally, features that contribute significantly to the top1-probability are selected.

#### 3.3.2. Sensitivity-Based Strategy

Some networks have several non-linear FC layers and it is not easy to measure their contributions. Consider the unrecognizability prediction task for example. First, we train a model with the backbone of EfficientNet-B0 (Tan and Le, 2019) and three FC layers with RELU to regress the unrecognizability score. Then, we adopt a sensitivity-based method to select features and the sensitivity can be obtained by gradients. Specifically, the input feature is *f*_*j*_, *j* = 1, 2, ...*N*_*d*_ and the FC layers with active function are represented by function *F*. The unrecognizability score *s* can be obtained by


(5) $$\begin{array}{r} s=F\left(f_{j}\right). \end{array}$$

The sensitivity of features can be described by


(6) $$\begin{array}{r} grad_{j}=abs\left(\partial s/\partial f_{j}\right). \end{array}$$

Equation (6) represents the importance of features through partial derivatives, which is widely used in sensitivity analysis and model interpreting (Garson, 1991; Dimopoulos et al., 1995). After obtaining the importance of features, the selected number is calculated. Then, the index of sorted features can be obtained through


(7) $$\begin{array}{r} ind_{j}=argsort\left(grad_{j}\right), \end{array}$$

where “*argsort*” means that sort the sequence and return corresponding index (it is the same with “*argsort*” in Algorithm 1). Finally, a selection operation is executed.

In contrast to directly merging all intelligibility features with distortion features, fusing features with lower dimension after feature selection exhibits better performance and generalization ability during the test process. Different from attention mechanism, the proposed feature selection strategy can reduce the dimension of the intelligibility feature and does not need any additional module or further training.

## 4. Experiments

### 4.1. Datasets

In our experiments, five image quality datasets with authentic distortions are adopted, including KonIQ-10k (Hosu et al., 2020), Smartphone Photography Attribute and Quality (SPAQ) (Fang et al., 2020), LIVE in the Wild Image Quality Challenge (LIVEW) (Ghadiyaram and Bovik, 2016), CID2013 (Virtanen et al., 2015), and BID (Ciancio et al., 2011). Specifically, the KonIQ-10k dataset has 10,073 labeled images selected from a massive public database YFCC100M (Thomee et al., 2016), and the labels are obtained from 1.2 million ratings. The SPAQ dataset contains 11,125 labeled images obtained from 66 smartphones with exchangeable image file format data tags and rich opinion annotations. The annotations include MOS, attribute scores (such as brightness, noisiness, and sharpness) as well as scene category labels. LIVEW contains 1,162 labeled images and CID2013 contains 480 images from eight scenes. Different from the other four datasets, the BID dataset focuses on blur images and contains 586 images.

### 4.2. Implementation and Evaluation Protocol

In our experiments, the distortion network adopts the backbone of EfficientNet-B0 and the intelligibility network for the image recognition task is EfficientNet-B0 as well. EfficientNet-B0 consists of one convolutional layer followed by seven mobile inverted bottleneck modules, and then another convolutional layer followed by global average pooling. EfficientNet-B0 has an input size of 224 × 224 and 5.3 M parameters, and the dimension of its output feature is 1280. Network for scene classification task is ResNet-18 (He et al., 2016), and object detection is Faster-RCNN (Ren et al., 2017) with ResNet50-FPN (Lin et al., 2017) backbone. The instance segmentation task is DeeplabV3+ (Chen et al., 2018) with the backbone of ResNet101. During the training process, SGD optimizer with initial learning-rate 0.03 is utilized (we train FC layers first and then utilize warm-up strategy when training the distortion backbone). For all of our experiments, we first resize images into 244 × 244, then we randomly crop them to 224 × 224 with a randomly horizontal flip to augment training images. During the test process, we directly resize test images into 224 × 224 and then predict once, which is more efficient in real applications. We tried different selection ratios of 1, 5, 20, and 50%. The final selection ratio of the recognition task, class task, detection task, segmentation task, and unrecognization task are 5, 5, 20, 50, and 50%, respectively.

Our evaluation criteria are two widely used correlation coefficients: Pearson's linear correlation coefficient (PLCC) and Spearman's rank order correlation coefficient (SRCC).

### 4.3. Performance Comparison

This paper aims to propose a highly generalizable NR-IQA model, thus we train our model in one dataset and then test on other datasets directly without doing any fine-tuning. For comparison, we also re-train some popular handcrafted feature-based methods, such as BRISQUE, CORNIA, HOSA, and deep learning-based methods, including DBCNN (Zhang W. et al., 2020), MetaIQA (Zhu et al., 2020), and WaDIQaM-NR (Bosse et al., 2017) (codes are publically available) with the same setting. All results trained on KonIQ-10k are shown in Table 1. The middle group in Table 1 shows deep learning-based methods, and the results of methods without public codes are obtained from the original papers. The bottom group shows our results.

**Table 1:** Pearson's linear correlation coefficient (PLCC)/Spearman's rank order correlation coefficient (SRCC) results of cross-dataset test.

**PLCC/SRCC**	**KonIQ-10k**	**SPAQ**	**LIVEW**	**CID**	**BID**
BIQI Moorthy and Bovik, 2010	0.637/0.595	0.622/0.661	0.492/0.471	0.612/0.599	0.478/0.493
NFERM Gu et al., 2014	0.725/0.689	0.697/0.711	0.551/0.540	0.708/0.680	0.529/0.530
BRISQUE Mittal et al., 2012	0.689/0.647	0.660/0.682	0.576/0.554	0.553/0.533	0.589/0.597
BLINDSII Saad et al., 2012	0.440/0.447	0.466/0.460	0.331/0.319	0.278/0.301	0.393/0.401
GWH-GLBP Li et al., 2016	0.549/0.514	0.614/0.628	0.464/0.435	0.071/0.002	0.477/0.483
FISBLIM Gu et al., 2013	0.375/0.347	0.566/0.569	0.376/0.289	-0.219/-0.234	0.392/0.344
CORNIA Ye et al., 2012	0.773/0.738	0.727/0.766	0.672/0.639	0.599/0.538	0.692/0.688
HOSA Xu et al., 2016	0.791/0.761	0.743/0.771	0.677/0.652	0.684/0.664	0.694/0.679
NSSADNN Yan et al., 2019	/	/	0.813/0.745*	0.825/0.748*	/
MEON Ma et al., 2017	/	/	0.693/0.688*	0.703/0.701*	/
BIECON Kim and Lee, 2017	/	/	0.613/0.595*	0.620/0.606*	/
DeepRN (ResNet101) Varga et al., 2018	0.880/0.867	/	0.750/0.726	/	/
DeepBIQ (InceptionV2) Bianco et al., 2018	0.911/0.907	/	0.821/0.804	/	/
HyperNet Su et al., 2020	0.917/**0.906**	0.843/0.846^+^	NA/0.785	0.808/0.782^+^	NA/**0.819**
MetaIQA Zhu et al., 2020	0.876/0.846	0.804/0.822	0.748/0.716	0.726/0.682	0.740/0.738
WaDIQaM-NR Bosse et al., 2017	0.657/0.631	0.675/0.702	0.521/0.523	0.584/0.495	0.499/0.538
DBCNN Zhang W. et al., 2020	0.892/0.868	0.827/0.836	0.802/0.775	0.788/0.758	0.769/0.769
**Our Results**					
**IE-IQA (** *w/ recognition task)*	**0.921**/0.900	**0.863/0.859**	**0.839/0.829**	0.815/0.788	**0.822**/0.817
**IE-IQA (** *w/ classification task)*	0.920/0.900	0.862/0.858	0.835/0.828	0.818/0.795	0.819/0.813
**IE-IQA (** *w/ detection task)*	**0.921/**0.901	0.862/0.857	0.835/0.826	0.819/0.800	0.816/0.810
**IE-IQA (** *w/ segmentation task)*	0.917/0.900	0.862/0.857	0.825/0.826	**0.827/0.801**	0.812/0.809
**IE-IQA (** *w/ unrecognization task)*	0.920/ 0.902	**0.863**/0.858	0.835/**0.829**	0.819/0.794	0.816/0.813

The model is trained on 80% images of KonIQ-10k and directly tested on rest 20% KonIQ-10k images and other datasets. Results with “*” are obtained after fine-tuning on the dataset and reported in original papers. Results with NA of HyperNet (only three datasets) are reported in original papers (Su et al., 2020) and results with “+” are obtained from the released model. Best results are in bold.

From Table 1, we can observe that our framework with five intelligibility tasks can consistently achieve the best cross-dataset performance for most cases. It should be emphasized that our models are only trained with KonIQ-10k (80% images) and directly tested on other datasets without any fine-tuning. Though NSSADNN, MEON, and BIECON made fine-tuning on the target dataset, our generalization performance can still maintain a significant advantage.

**Figure 4:**
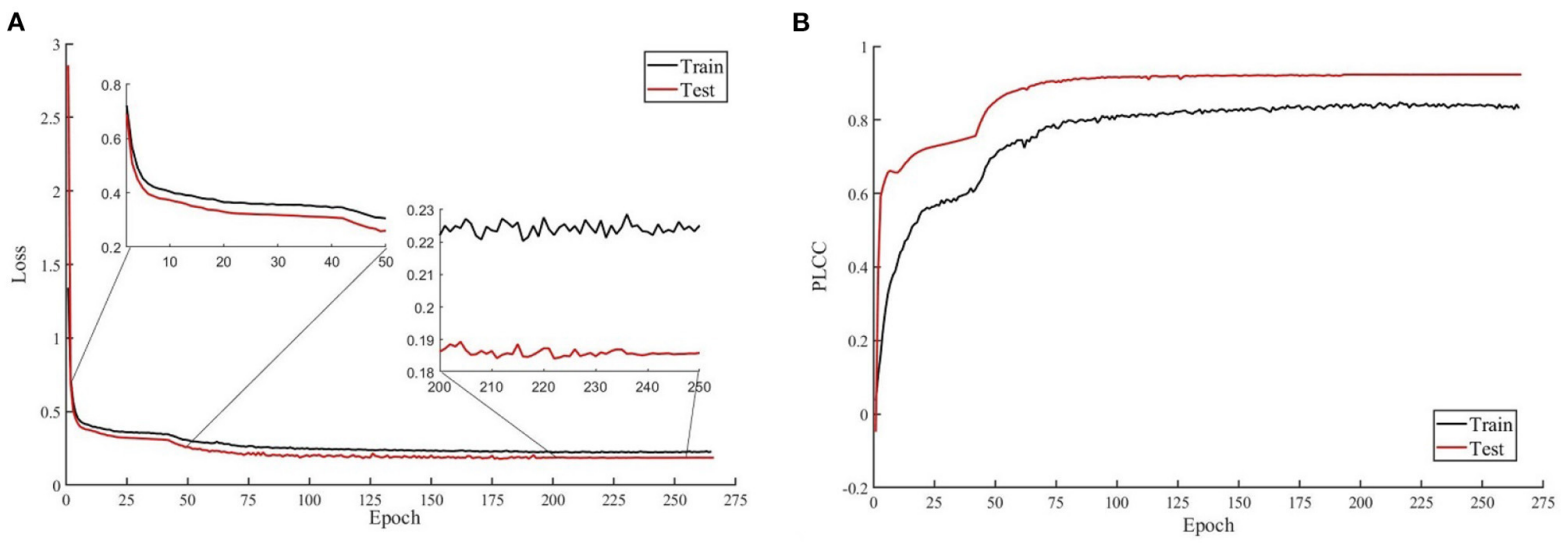
Loss and Pearson's linear correlation coefficient (PLCC) during training and test. **(A)** Loss of training and test. Two enlarged subfigures shows results of epochs 2–50 and epochs 200–250. **(B)** PLCC of training and test. The model is trained with recognition task on KonIQ-10k.

Efficient-B0 has 5.3M parameters, which is less than ResNet18 (11.7 M parameters, the backbone of MetaIQA), ResNet50 (26 M parameters, the backbone of HyperNet), and ResNet101 (44.5 M parameters, the backbone of DeepRN). Efficient-B0 is easy to converge, and we show the loss and PLCC results during training and test in Figure 4. We can observe from Figure 4 that the test loss decreases with the training loss and the test performance increases with training performance. This means that the network is trained without overfitting.

To make a further comparison, we also train our methods on SPAQ and perform cross-dataset tests on the other four datasets. The results are shown in Table 2.

**Table 2:** Pearson's linear correlation coefficient (PLCC)/Spearman's rank order correlation coefficient (SRCC) results of cross-dataset test.

**PLCC/SRCC**	**SPAQ**	**KonIQ-10k**	**LIVEW**	**CID**	**BID**
NFERM Gu et al., 2014	0.832/0.823	0.455/0.447	0.591/0.542	0.437/0.342	0.578/0.570
BRSIQUE Mittal et al., 2012	0.833/0.822	0.446/0.433	0.593/0.553	0.499/0.504	0.589/0.578
CORNIA Ye et al., 2012	0.867/0.859	0.532/0.516	0.663/0.621	0.552/0.465	0.676/0.673
HOSA Xu et al., 2016	0.873/0.866	0.559/0.534	0.682/0.650	0.593/0.536	0.681/0.670
Baseline Fang et al., 2020	0.909/0.908	0.532/0.523^+^	0.564/0.517^+^	0.518/0.569^+^	0.574/0.566^+^
MT-S Fang et al., 2020	**0.921/0.917**	0.486/0.485^+^	0.539/0.493^+^	0.342/0.389^+^	0.530/0.529^+^
HyperNet Su et al., 2020	0.917/0.915	0.679/0.645	0.695/0.680	0.624/0.585	0.648/0.647
MetaIQA Zhu et al., 2020	0.871/0.870	0.722/0.686	0.765/0.731	0.737/0.695	0.743/0.735
**Our Results**					
**IE-IQA (** *w/ recognition task)*	0.918/0.913	0.768/0.710	0.779/0.764	0.743/0.713	0.744/0.742
**IE-IQA (** *w/ classification task)*	0.917/0.915	0.761/0.720	0.764/0.758	0.737/0.702	0.737/0.737
**IE-IQA (** *w/ detection task)*	0.920/0.916	**0.777/0.728**	**0.782/0.772**	0.742/0.702	**0.748/0.749**
**IE-IQA (** *w/ segmentation task)*	0.918/0.914	0.775/0.724	0.781/0.768	**0.752/0.737**	0.744/0.746
**IE-IQA (** *w/ unrecognization task)*	0.920/0.916	0.770/0.721	0.774/0.764	**0.752**/0.725	0.747/0.746

*The model is trained on 80% images of Smartphone Photography Attribute and Quality (SPAQ) and directly tested on rest 20% SPAQ images and other datasets. Results with “+” are obtained from the released model. HyperNet are retrained with image size of 244 × 244. Best results are in bold*.

The model “Baseline” in Fang et al. (2020) means the baseline model (ResNet50) and “MT-S” means the model jointly trained with MOS and scene labels (The SPAQ dataset has scene category labels). We can observe that compared to MT-S, our method can achieve similar performance on the training dataset. However, by combining intelligibility features, the generalization performance of the proposed method is apparently much better.

Comparing Table 2 with Table 1, we can observe that models trained on KonIQ-10k have better cross-dataset performance. One possible reason is the source of images. The SPAQ dataset is obtained from smartphones only, while the image sources of KonIQ-10k are more diversified. Another possible reason is that the image size of the SPAQ dataset is very large (4000 × 3000 is common) and our model has an input size of 224 × 224. Small size input may lose much information and the interpolation algorithm may bring new distortions.

Another phenomenon observed from Tables 1, 2 is that the proposed method achieves slightly worse generalization performance on the BID/CID databases than the other three datasets. The BID dataset focuses on blur images and the CID dataset consists of limited scenes of images (eight scenes). This may lead to a more pronounced distribution discrepancy between CID/BID and the training datasets.

Though our metric aims to achieve high generalization ability, we still make further experiments on intra-dataset tests. The results are listed in Table 3. We can summarize from Table 3 that our metric can achieve state-of-the-arts intra-dataset performance. Though HyperNet achieves better performance for some cases, it needs to evaluate crop 25 patches during evaluating, costing much more time than the proposed metric. For example, when evaluating 1,000 images with the resolution of 1024 × 768 (batchsize = 1, using one TITANXp GPU and Intel Xeon E5-2630V4 CPU), HyperNet costs 2,040 s, while the proposed metric only costs 84 s.

**Table 3:** Pearson's linear correlation coefficient (PLCC)/Spearman's rank order correlation coefficient (SRCC) results on intra-dataset tests.

**Dataset**	**KonIQ-10k**	**SPAQ**	**LIVEW**	**CID2013**	**RBID**
NFERM Gu et al., 2014	0.725/0.689	0.832/0.823	0.562 /0.517	0.825/0.823	0.585/0.559
BRISQUE Mittal et al., 2012	0.689/0.647	0.833/0.822	0.574/0.557	0.810/0.814	0.617/0.594
CORNIA Ye et al., 2012	0.773/0.738	0.867/0.859	0.692/0.655	0.822/0.803	0.712/0.695
HOSA Xu et al., 2016	0.791/0.761	0.873/0.866	0.703/0.667	0.835/0.833	0.716/0.684
NSSADNN Yan et al., 2019	/	/	0.813^*^/0.745^*^	0.825^*^/0.748^*^	/
MEON Ma et al., 2017	/	/	0.693^*^/0.688^*^	0.703^*^ / 0.701^*^	/
BIECON Kim and Lee, 2017	/	/	0.613^*^/0.595^*^	0.620^*^/0.606^*^	/
Baseline Fang et al., 2020	0.908/0.889	0.909^*^/0.908^*^	0.825/0.794	0.876/0.881	0.802/0.794
WaDIQaM-NR Bosse et al., 2017	0.805^*^/0.797^*^	/	0.680^*^/0.671^*^	0.729^*^/0.708^*^	0.742^*^/0.725^*^
HyperNet Su et al., 2020	0.917^*^/0.906^*^	0.914/0.909	**0.882** ^*^ **/0.859** ^*^	/	0.878^*^/0.869^*^
DBCNN Zhang W. et al., 2020	0.892/0.868	0.915^*^/0.911^*^	0.869^*^/0.851^*^	/	0.859^*^/0.845^*^
MetaIQA Zhu et al., 2020	0.887^*^/0.850^*^	0.871/0.870	0.835^*^/0.802^*^	0.784^*^/0.766^*^	0.777/0.746
**IE-IQA (** *w/ recognition task)*	**0.921/**0.900	**0.918/0.913**	0.868/0.838	**0.934/0.934**	0.838/0.837

Results with * are obtained from published papers. Other results are obtained from retrained model. Best results are marked in bold.

**Figure 5:**
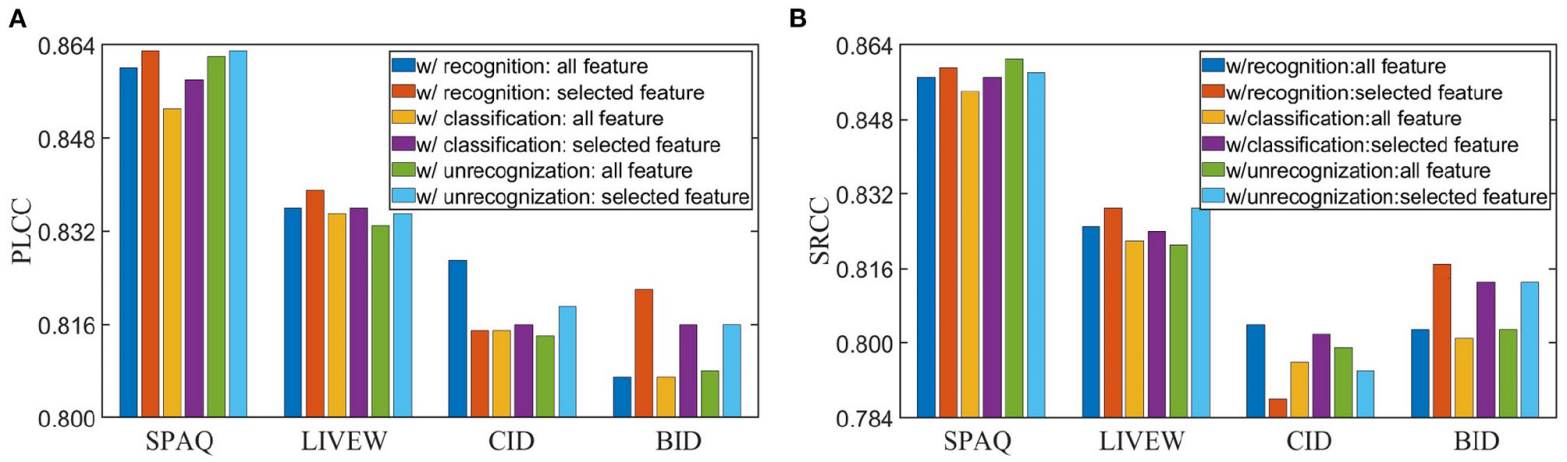
Performance comparison for different tasks with/without feature selection strategy on KonIQ-10k. **(A)** Pearson's linear correlation coefficient (PLCC) results. **(B)** Spearman's rank order correlation coefficient (SRCC) results. The recognition and classification task utilize contribution-based strategy, and the unrecognizability task utilizes gradient-based strategy.

To explore how feature selection strategy affects prediction results, we make a comparison of the results with/without feature selection strategy, and show them in Figure 5. The results show that removing noisy features and utilizing features having significant influence on final predictions tend to achieve higher performance and better generalization ability with only one exception (recognition task) on the CID dataset. One possible reason is that the CID dataset has only eight specific scenes and many images in CID contain the same objects. In this situation, selected features may not provide rich distinguished information for evaluating quality of images with similar contents.

**Figure 6:**
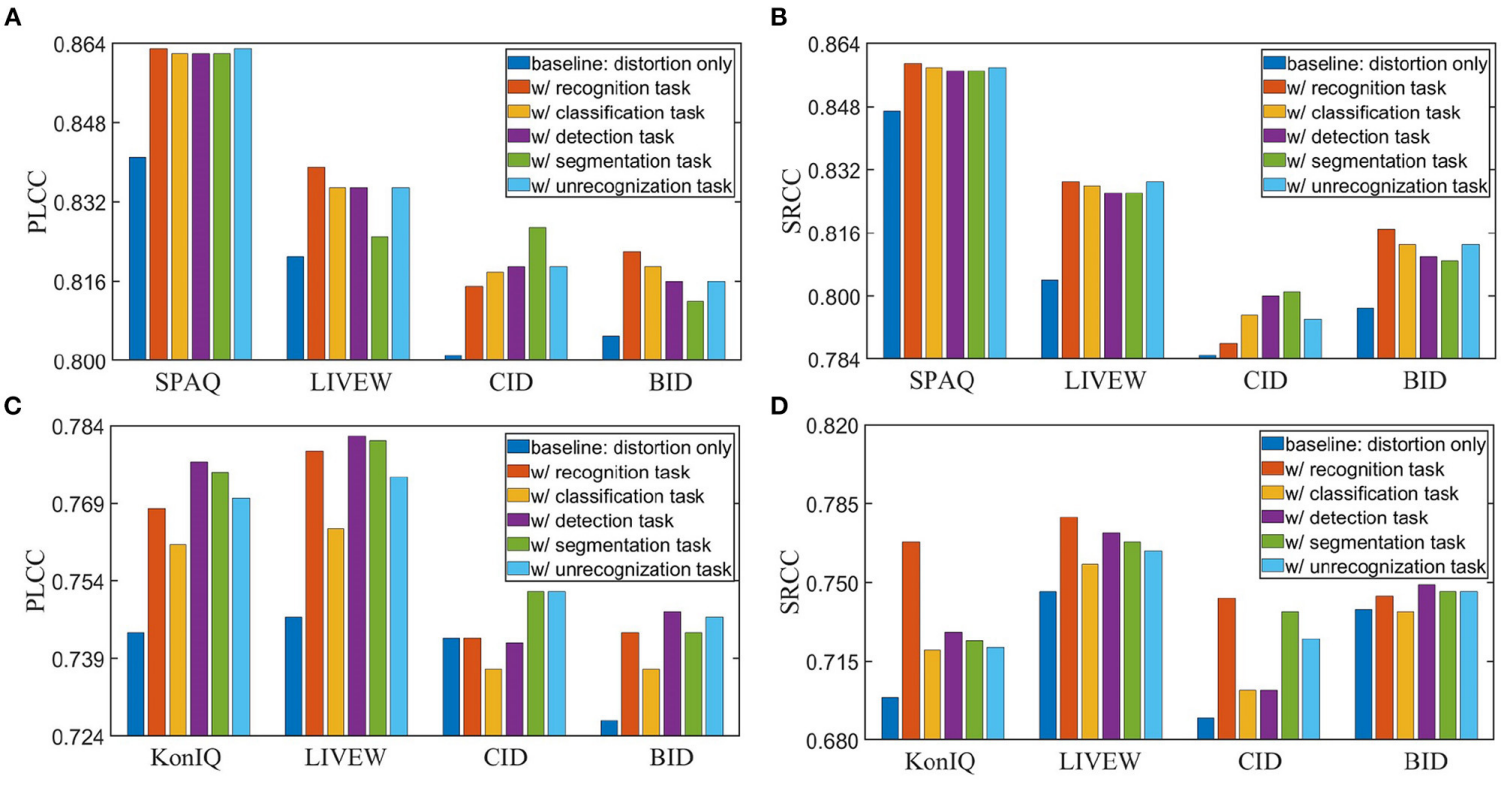
Ablation study of intelligibility enriched IQA (IE-IQA). **(A)** Pearson's linear correlation coefficient (PLCC) results of models trained with 80% KonIQ-10k. **(B)** Spearman's rank order correlation coefficient (SRCC) results of models trained with 80% KonIQ-10k. **(C)** PLCC results of models trained with 80% Smartphone Photography Attribute and Quality (SPAQ). **(D)** SRCC results of models trained with 80% SPAQ.

To demonstrate the effectiveness of intelligibility features, we make ablation studies and show the results in Figure 6. The baseline means the model with distortion backbone alone. From Figure 6, we can observe that intelligibility features do improve both performance and generalization ability. Therefore, it is necessary to combine both intelligibility aspect and distortion aspect in IQA metrics.

During the training process, the distortion network loads the pre-trained model, and some semantic information and intelligibility features may have already existed in the pre-trained model. To further investigate the effects of original intelligibility features on the distortion network, we train the distortion network from scratch. Then we fuse the intelligibility network with the distortion network. The results are shown in Tables 4, 5. From the tables, we can observe that the introduced intelligibility network still benefits the performance of the whole framework even the distortion network is not pre-trained.

**Table 4:** Results of training the distortion network from scratch on 80% KonIQ-10k.

**PLCC**	**KonIQ-10k(20%)**	**SPAQ**	**LIVEW**	**CID**	**BID**
Only distortion	0.784	0.756	0.638	0.676	0.645
W/recognition	0.814	**0.812**	**0.689**	**0.714**	**0.706**
W/classification	0.814	0.763	0.653	0.695	0.659
W/detection	0.812	0.740	0.644	0.682	0.640
W/segmentation	**0.826**	0.758	0.676	0.688	0.684
W/unrecognization	0.811	0.749	0.651	0.691	0.666

*Best results are in bold*.

**Table 5:** Results of training the distortion network from scratch on 80% Smartphone Photography Attribute and Quality (SPAQ).

**PLCC**	**SPAQ(20%)**	**KonIQ-10k**	**LIVEW**	**CID**	**BID**
only distortion	0.878	0.568	0.605	0.665	0.598
w/recognition	0.883	0.591	**0.628**	**0.702**	0.628
w/classification	**0.884**	0.585	0.625	0.695	**0.631**
w/detection	0.882	**0.598**	0.626	0.698	0.623
w/segmentation	0.883	0.592	0.627	0.697	0.629
w/unrecognization	0.881	0.585	0.621	0.686	0.615

*Best results are in bold*.

**Figure 7:**
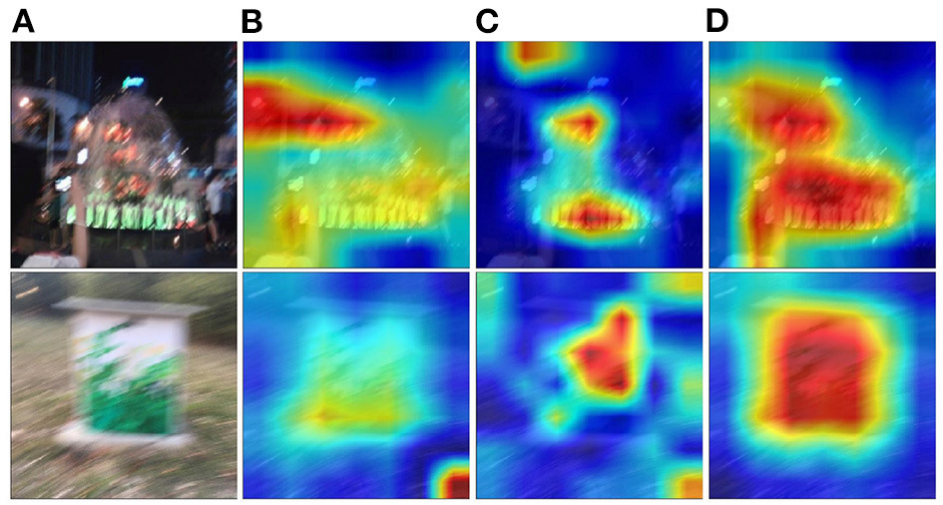
Visualization results of Grad-CAM. **(A)** Original images; **(B)** heat-maps of the baseline model; **(C)** heat-maps of the intelligibility network; **(D)** heat-maps of proposed model with image recognition task.

To explore how intelligibility affects quality assessment results intuitively, we utilize the method of Grad-CAM (Selvaraju et al., 2017) to investigate which area of an image affects the prediction most. Examples are shown in Figure 7, where red areas have more conspicuous influence to the prediction than blue areas. As shown in Figure 7, the intelligibility features do play an important role in the quality assessment. The baseline model with distortion network only (Figure 6B) cannot effectively locate salient objects which people may pay attention to. The intelligibility features (Figure 6C) alone mainly focus on relatively local regions and cannot well utilize global information of images. In contrast, the proposed model (Figure 6D) not only meticulously locate salient objects (important for intelligibility), but also pay more attention to wider areas, which catches global information. It is widely acknowledged that both global and local information are vital to IQA metrics (Fang et al., 2018); hence, from this point of view, it is not hard to understand that by combining the intelligibility features, our model can achieve better performance.

## 5. Conclusions

In this paper, we first analyzed the relation between intelligibility and image quality. The results reveal that intelligibility is indicative of image quality. Therefore, we proposed a new framework, i.e., Intelligibility-Enriched-IQA, to combine intelligibility with conventional distortion measure. Feature selection strategy was proposed to select the most important intelligibility features, which alleviates negative transfer and avoids damaging highly generalizable features. Extensive experimental results show the effectiveness of proposed method, and our model achieves state-of-the-art performance in terms of the generalization ability. These results demonstrate that introducing intelligibility is a promising way in building highly generalizable IQA metrics.

## Data Availability Statement

The datasets presented in this study can be found in online repositories. The names of the repository/repositories and accession number(s) can be found in the article/supplementary material.

## Author Contributions

TS and LL contributed to conception and design of the study. TS performed the experiment and wrote the first draft of the manuscript. LL, HZ, and JQ wrote sections of the manuscript. All authors contributed to manuscript revision, read, and approved the submitted version.

## Funding

This work was supported in part by the National Natural Science Foundation of China under Grants 62171340, 61771473, 61991451, and 61379143, the Key Project of Shaanxi Provincial Department of Education (Collaborative Innovation Center) under Grant 20JY024, the Fundamental Research Funds for the Central Universities under Grant JBF211902, the Science and Technology Plan of Xi'an under Grant 20191122015KYPT011JC013, the Natural Science Foundation of Jiangsu Province under Grants BK20181354 and BK20200649, and the Six Talent Peaks High-level Talents in Jiangsu Province under Grant XYDXX-063.

## Conflict of Interest

The authors declare that the research was conducted in the absence of any commercial or financial relationships that could be construed as a potential conflict of interest.

## Publisher's Note

All claims expressed in this article are solely those of the authors and do not necessarily represent those of their affiliated organizations, or those of the publisher, the editors and the reviewers. Any product that may be evaluated in this article, or claim that may be made by its manufacturer, is not guaranteed or endorsed by the publisher.
